# Death of a Noted Southern Physician

**Published:** 1871-05

**Authors:** 


DEATH OF A NOTED SOUTHERN PHYSICIAN.—Dr. R. B. Wellford, a prominent physician, and for many years a Professor of Materia Medica in the Virginia Medical Colleges, died Dec. 29th, in Richmond, aged 74.

